# Characteristics of short-term acute care hospitals that experienced a ransomware attack from 2016 to 2021

**DOI:** 10.1093/haschl/qxad037

**Published:** 2023-08-28

**Authors:** Claire C McGlave, Sayeh S Nikpay, Carrie Henning-Smith, Katie Rydberg, Hannah T Neprash

**Affiliations:** Division of Health Policy and Management, University of Minnesota School of Public Health, Minneapolis, MN 55455, United States; Division of Health Policy and Management, University of Minnesota School of Public Health, Minneapolis, MN 55455, United States; Division of Health Policy and Management, University of Minnesota School of Public Health, Minneapolis, MN 55455, United States; Division of Health Policy and Management, University of Minnesota School of Public Health, Minneapolis, MN 55455, United States; Division of Health Policy and Management, University of Minnesota School of Public Health, Minneapolis, MN 55455, United States

**Keywords:** cybersecurity, health information technology, hospital

## Abstract

As cybercrime increasingly targets the health care sector, hospitals face the growing threat of ransomware attacks. Ransomware is a type of malicious software that prevents users from accessing their electronic systems—demanding payment to restore access. In response, momentum is gathering to enact policy that will help hospitals strengthen their cybersecurity defenses. However, to design effective policy, it is crucial to understand the characteristics of hospitals associated with the risk of ransomware attack. In this paper, we compare the characteristics of ransomware-attacked and non-attacked short-term acute care hospitals in the United States. Using data from the American Hospital Association's Annual Survey and the Healthcare Cost Report Information System, we found that ransomware-attacked hospitals were larger, had higher net operating revenue, were more likely to be financially profitable, and more likely to provide trauma, emergency, and obstetric care than non-attacked hospitals. Measures of information technology sophistication did not vary between ransomware-attacked and non-attacked hospitals. These results can be used to tailor policy interventions in order to most effectively respond to and prevent cybercrime in health care.

## Introduction

From 2016 to 2021, the annual number of ransomware attacks on health care delivery organizations in the United States more than doubled.^1^ Ransomware attacks are designed to disrupt business operations in order to motivate the prompt payment of ransom demands by victims. This form of cyberattack appears to be particularly disruptive for hospitals, where nearly 75% of ransomware attacks affected patient care in some way.^1^ Documented disruptions included ambulance diversion, electronic system downtime, and delays in scheduled care.^1,2^ Notably, ransomware attacks on hospitals also affect care at neighboring hospitals, with evidence suggesting crowding at nearby emergency departments when one hospital in a market experiences an attack.^3^

Ransomware attacks are potentially harmful to patients in two ways. First, many ransomware attacks result in the exposure of personal health information. From 2016 to 2021, ransomware attacks on health care delivery organizations exposed the personal health information of 42 million patients.^1^ Second, care disruptions caused by ransomware attacks may harm patient safety and negatively impact health outcomes. For example, without access to an electronic health record, adverse drug events may occur more frequently. Similarly, a patient presenting with a heart attack whose ambulance must divert to a more distant hospital may experience a delay in time-to-treatment,^3^ potentially resulting in higher morbidity and mortality.

Responding to this growing threat of ransomware attacks in health care, policymakers and regulators are proposing strategies to strengthen the cybersecurity defenses of hospitals.^4–6^ Whether these proposals take the form of legislative or regulatory activity remains uncertain, but it seems likely that hospitals will soon face minimum cybersecurity requirements. These requirements will likely mandate the use of technologies designed to minimize unauthorized access to electronic systems, such as multifactor authentication, email protection systems, endpoint protection systems, and other recommended technological investments.^7^ This represents a major change from the status quo, since the average hospital currently invests less than ten percent of its information technology (IT) budget in cybersecurity.^8^

To craft evidence-based policy incentivizing hospital cybersecurity improvements of this magnitude, policymakers need a better understanding of hospital characteristics associated with a greater risk of ransomware attack. Three questions of interest stand out—the answers to which will help target policy requirements and potential financial support. First, are hospitals that experience ransomware attacks more likely to be struggling financially (ie, lower net operating revenue and less likely to be profitable)? Second, do ransomware-attacked hospitals play a different role in their local health care delivery system than non-attacked hospitals? And third, do ransomware-attacked hospitals have less developed IT systems, indicating potential vulnerabilities? In this paper, we answer these questions using a variety of data sources on hospital characteristics, linked to a database of ransomware attacks on US hospitals between 2016 and 2021.

## Data and methods

This was a secondary analysis of data on hospital characteristics from the American Hospital Association (AHA) Annual Survey Database,^9^ the AHA Annual Survey Information Technology Supplement, the Healthcare Cost Report Information System (HCRIS), and the College of Healthcare Information Management Executives (CHIME) Digital Health Most Wired survey. To identify hospitals that did and did not experience a ransomware attack during our study period (2016–2021), we linked these datasets to the Tracking Healthcare Ransomware Events and Traits (THREAT) database. The THREAT database (described in greater detail and validated elsewhere^1^) combines proprietary data provided by HackNotice (a cybersecurity threat intelligence company that helps businesses identify and respond to attacks) with data from the US Department of Health and Human Services (HHS) Office of Civil Rights Data Breach Portal. The latter includes publicly available information that is collected when Health Insurance Portability and Accountability Act–covered entities report breaches of protected health information, as mandated by the Health Information Technology for Economic and Clinical Health Act of 2009.^10^ We limited our sample to US short-term acute care hospitals in the AHA data that reported providing general medical and surgical services. This sample restriction eliminated a modest number of specialty hospitals, in an attempt to compare hospitals delivering similar services. We assessed all hospital characteristics in 2019 (regardless of ransomware attack date), to avoid detecting differences reflective of secular changes among all hospitals over time. Using hospital ZIP codes from the AHA survey, we classified each as rural vs urban using the Federal Office of Rural Health Policy methodology^11^ and assigned each to a Hospital Referral Region (HRR).^12^

Information on the financial position of hospitals included net patient revenue and all-payer profit margin, both from HCRIS.^13,14^ We also used the AHA survey^15^ to assess whether each hospital belonged to a larger health system (which may indicate greater financial reserves, due to the higher service prices that system-affiliated hospitals command from commercial insurers^16,17^) and was a nonprofit organization (with different rules around the reinvestment of profits, compared with for-profit and government hospitals).

Information on each hospital's role in their local health care delivery system included whether the hospital was a level 1 or 2 trauma center, whether the hospital operated an emergency room, and whether the hospital operated an obstetric unit. Similarly, indicators for Critical Access Hospitals, Sole Community Hospitals, Rural Referral Centers, and hospitals in rural areas (many, but not all, of which have the former distinctions) identify facilities in communities where there are few alternative inpatient treatment locations nearby. Finally, we constructed a market share variable for each hospital, equal to its share of total annual inpatient admissions within its local market (defined as Hospital Referral Region^18^).

Information on each hospital's IT sophistication included whether they were deemed a “Most Wired” hospital by CHIME (ie, a designation billed as a “digital health check-up for health care organizations”).^19,20^ We also used the AHA IT supplement to quantify whether hospitals had a comprehensive, basic, or sub-basic electronic health record (EHR)—a composite measure indicating the existence of electronically maintained patient demographic information, physician notes, nursing assessments, problem lists, medication lists, discharge summaries, and laboratory/imaging reports.^21,22^

To compare characteristics of ransomware-attacked and non-attacked hospitals, we used logistic (ordinary least squares) regression for binary (continuous) variables. Marginal effects were computed using the Stata version 17.0 margins command (StataCorp). All analyses used Huber-White robust standard errors to assess statistical significance, defined as *P* < .05.

## Results

**Figure 1.**
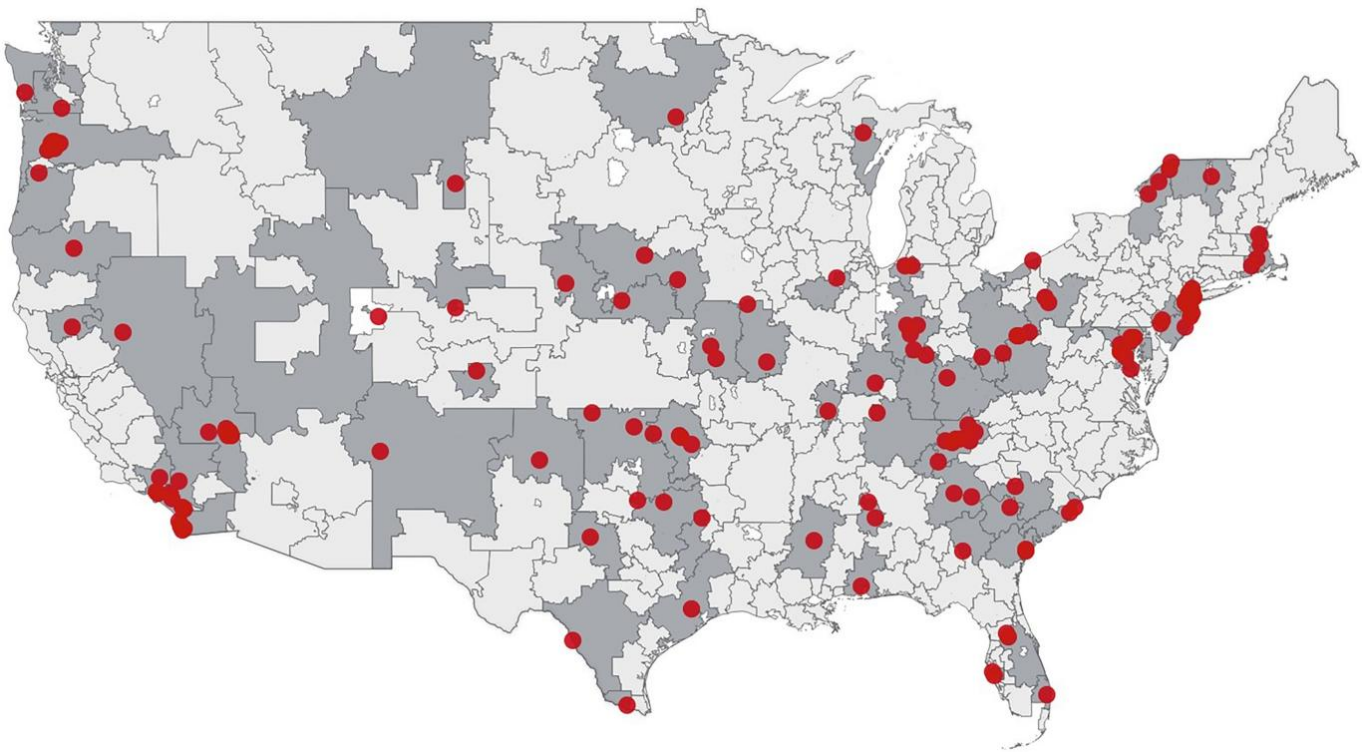
Location of ransomware-attacked hospitals, 2016-2021. Red dots represent attacked hospitals (n = 163). Shaded areas represent Hospital Referral Regions that experienced a ransomware attack between 2016 and 2021 (n = 72).

Our sample included 4,531 short-term acute care hospitals providing general medical and surgical services, 163 (3.7%) of which experienced a ransomware attack between January 2016 and December 2021. Of the 306 HRRs in the United States, 72 (23.5%) contained a ransomware-attacked hospital during the study period (Figure 1).

Financial characteristics of ransomware-attacked and non-attacked hospitals differed considerably, with higher average net operating revenue among attacked hospitals ($361 million vs $222 million; *P* < .01; Table 1) and a larger share of attacked hospitals reporting positive all-payer profit margins (77.1% vs 69.8%; *P* = .03) Attacked and non-attacked hospitals were equally likely to belong to a larger health system (69.9% vs 67.5%; *P* = .51) and less likely to be a nonprofit hospital (77.3% vs 86.0%; *P* < .01) rather than a for-profit or government-owned hospital.

**Table 1.** Characteristics of ransomware-attacked hospitals and non-attacked hospitals, 2016–2021.

Hospital characteristics	Attacked (n = 163)	Non-attacked (n = 4,368)	*P*
Financial characteristics			
Net operating revenue,^a^ mean	$361 million	$222 million	<.01
Positive profit margin,^a^ %	77.1	69.8	.03
System membership, %	69.9	67.5	.51
Nonprofit status, %	77.3	86.0	<.01
Role in delivery system			
Level 1 or 2 trauma center, %	23.9	12.9	<.01
Operates an emergency room, %	80.4	73.2	.02
Operates an obstetric unit, %	68.7	47.1	<.01
Share of total admissions in HRR, mean %	7.9	6.7	.14
Critical Access Hospital, %	6.1	30.5	<.01
Sole Community Hospital, %	9.8	6.6	.17
Rural Referral Center, %	7.4	7.0	.86
Rurally located, %	26.4	48.9	<.01
Information technology infrastructure			
Sub-basic EHR capabilities,^b^ %	9.4	7.1	.45
Basic EHR capabilities,^b^ %	17.7	18.5	.85
Comprehensive EHR capabilities,^b^ %	72.9	74.4	.74
Most Wired Hospital status, %	31.3	33.2	.62

Abbreviations: EHR, electronic health record; HRR, Hospital Referral Region.

This table presents comparisons of characteristics between ransomware-attacked hospitals and hospitals that did not experience a ransomware attack from 2016 through 2021. Each row presents results from a logistic (ordinary least squares) regression of a binary (continuous) hospital characteristic on an indicator for whether the hospital had experienced a ransomware attack.

a Denotes a sample size of 157 attacked hospitals and 3751 non-attacked hospitals.

b Denotes a sample size of 96 attacked hospitals and 2550 non-attacked hospitals.

Ransomware-attacked hospitals also had characteristics indicative of a different role within their local health care delivery system than non-attacked hospitals. Attacked hospitals were more likely than non-attacked hospitals to be level one or two trauma centers (23.9% vs 12.9%; *P* < .01), more likely to operate an emergency room (80.4% vs 73.2%; *P* = .02), and more likely to operate an obstetric unit (68.7% vs 47.1%; *P* < .01). Attacked hospitals did not differ from non-attacked hospitals in the share of their local hospital market's annual inpatient admission volume (7.9% vs 6.7%; *P* < .14). Ransomware-attacked hospitals were less likely than non-attacked hospitals to be Critical Access Hospitals (6.1% vs 30.5%; *P* < .01), but equally likely to be Sole Community Hospitals (9.8% vs 6.6%; *P* = .17) and Rural Referral Centers (7.4% vs 7.0%; *P* = .86). Overall, ransomware-attacked hospitals were less likely than non-attacked hospitals to be located in rural areas (26.4% vs 48.9%; *P* < .01).

IT capabilities did not differ between ransomware-attacked hospitals and their non-attacked counterparts. Attacked and non-attacked hospitals were equally likely to have sub-basic (9.4% vs 7.1%; *P* = .45), basic (17.7% vs 18.5%; *P* = .85), and comprehensive EHR capabilities (72.9% vs 74.4%; *P* = .74)—and equally likely to have earned “Most Wired” status (31.3% vs 33.2%; *P* = .62).

## Discussion

Between 2016 and 2021, ransomware attacks affected 3.7% of short-term acute care hospitals in the United States and 23.5% of hospital markets. Attacked hospitals had larger operating revenue and were more likely to provide trauma, emergency, and obstetric services in urban areas. The finding that nearly one in four hospital markets contains a ransomware-attacked hospital is particularly concerning, given emerging evidence that ransomware attacks generate spillover effects for nearby hospitals that may absorb displaced patient volume.^3^

Our findings provide the first answers to the three policy-relevant questions posed in the Introduction. On the question of how financial position differs between ransomware-attacked and non-attacked hospitals, we found evidence that the former group has higher net operating revenue and is more likely to be financially profitable. This may reflect some degree of targeting on the part of cybercriminals, perhaps selecting hospitals based on their perceived ability to pay a higher ransomware if the attack is successful. This type of targeting is consistent with the behavior of certain ransomware organizations known to operate against the health and public health sector.^23^

On the question of how ransomware-attacked and non-attacked hospitals differ in the role within their local health care delivery system, we found an indication that attacked hospitals are more likely to provide trauma, emergency, and obstetric care. Since these are patient populations where care delivery disruptions are potentially quite harmful, this finding may also reflect targeting by cybercriminals—if they are trying to identify targets with high motivation to pay a ransom quickly and restore operations. Conversely, our finding that a smaller share of ransomware-attacked hospitals were rurally located challenges the common perception that ransomware attacks are more likely to afflict underresourced rural hospitals.^24^ Despite lower rates of ransomware attacks in rural areas, additional research is needed to better understand the effects of ransomware attacks when they do affect rural facilities. Many rural hospitals are less equipped to handle these attacks if or when they occur due to a shortage of IT personnel in rural areas and smaller budgets for cybersecurity infrastructure.^25^ Rural residents—who are already in poorer health and have fewer financial resources than their urban counterparts—may have a more difficult time weathering a ransomware attack and traveling to an alternative site of care, if necessary.

On the question of IT sophistication, we found no statistically significant differences between ransomware-attacked and non-attacked hospitals. If IT sophistication is a proxy for cybersecurity preparedness, this is also a finding that may challenge the widespread assumption that ransomware attacks predominantly affect the most vulnerable hospitals with less developed IT defenses. This finding emphasizes the importance of increasing cybersecurity in all hospitals, and also illustrates a need for additional research and more nuanced measures to better understand vulnerability to cybersecurity threats.

This analysis was limited to hospital ransomware attacks appearing in the THREAT database, which likely represent an incomplete census of all ransomware activity against health care providers. However, we think that it is unlikely that hospital ransomware attacks are missing from the THREAT database, since they would need to receive zero news coverage and go unreported to the HHS in order to escape inclusion—and this is much more likely for small health care organizations (eg, small physician practices) compared with large entities like hospitals. Since we cannot observe attempted ransomware attacks that were unsuccessful, we cannot distinguish vulnerability to ransomware attacks (eg, fewer cybersecurity techniques used) from ransomware actors targeting specific types of hospitals. Further study is needed to understand factors that render hospitals vulnerable to cyberattack.

While we found that a small minority of hospitals were impacted by cyberattacks, such attacks are increasing in prevalence and require proactive action on the part of policymakers, regulators, and health care administrators. As policymakers craft legislation and regulation aimed at increasing cybersecurity defenses in health care,^4^ these findings suggest opportunities for targeting resources based on hospital characteristics.

## Supplementary Material

qxad037_Supplementary_Data
